# Effect of prior haptic virtual reality simulation on preclinical endodontic access cavity preparation

**DOI:** 10.3389/froh.2025.1673147

**Published:** 2025-11-05

**Authors:** Germán Sánchez-Herrera, Ola Deeb, Fernando José Alfaro-Ochoa, Martín Pérez-Leal, Cristina Palma-Carrió, Nicla Flacco

**Affiliations:** 1 Universidad Europea de Valencia, Faculty of Health Science, Department of Dentistry, Valencia, Spain; 2 School for Doctoral Studies and Research, Universidad Europea de Madrid, Madrid, Spain

**Keywords:** access cavity preparation, endodontics, simulation, haptic virtual reality, dental education

## Abstract

**Introduction:**

Haptic virtual reality simulation (HVRS) has emerged as a promising tool in dental education, supporting technical skill acquisition through interactive and feedback-rich environments. This study evaluated whether using HVRS as a preparatory step enhances student performance in endodontic access cavity preparation and explored students' perceptions of the simulation experience.

**Methods:**

Forty fourth-year dental students were assigned to two groups. The experimental group received HVRS training using the Simodont® Dental Trainer prior to conventional practice. The control group received only conventional training. All participants performed an endodontic access cavity on a premolar artificial tooth model, which was assessed using a validated evaluation rubric. Students in the experimental group also completed a post-simulation perception questionnaire.

**Results:**

The experimental group showed significantly higher scores in access cavity shape, roof removal, and internal form (*p* < 0.05). No difference was observed in damage to the pulpal floor. Most students reported that the simulator improved their understanding of internal anatomy and appreciated the opportunity to practice without risk. However, the majority disagreed that tactile realism matched that of a real handpiece.

**Conclusion:**

Integrating HVRS prior to conventional phantom-head training may improve specific technical aspects of endodontic access cavity preparation.

## 1 Introduction

Endodontic training in undergraduate dental curricula traditionally relies on artificial teeth mounted in phantom heads, where students practice access cavity preparation under faculty supervision. This approach provides a safe environment to acquire fundamental skills; however, it is limited by reduced standardization, variability in feedback, and difficulties in reproducing complex clinical scenarios (1). Moreover, errors made during initial attempts at endodontic procedures can negatively impact students' confidence and increase procedural anxiety (2).

Access cavity preparation represents a critical step for achieving correct treatment in endodontics (3). A well-designed access cavity facilitates canal location, preserves tooth structure, and provides straight-line access, thereby supporting both the efficiency and safety of root canal therapy (4, 5). For these reasons, preclinical education emphasizes accuracy and reproducibility during this phase, with the aim of reducing iatrogenic errors and improving student preparedness before transitioning to clinical care.

Haptic virtual reality simulation (HVRS) has emerged as a promising tool to complement conventional phantom-head training. By combining three-dimensional visualization with tactile feedback, HVRS offers students the opportunity to practice procedures in a standardized and interactive environment that allows for error detection and immediate correction (6, 7). In restorative dentistry, oral surgery, and pediatric dentistry, HVRS has been shown to improve manual dexterity, spatial awareness, and self-confidence (7–9). Recent syntheses suggest VR-based education can be at least as effective as conventional methods for several outcomes, while highlighting heterogeneity and research gaps (10).

In endodontics, HVRS has been applied to support skill development in access cavity preparation (11). Initial work with HVRS and augmented kinematic feedback demonstrated measurable gains in dental psychomotor skills (12). Early exposure to simulation may improve technical accuracy and reduce procedural anxiety while reinforcing three-dimensional anatomical understanding during the consolidation of fundamental motor skills (2, 13). In parallel, efforts to standardize assessment, such as structured rubrics that delineate key technical domains and common errors, aim to improve measurement reliability and comparability (14). Overall, the evidence remains limited, and the optimal placement of HVRS within the curriculum, whether as an alternative or as a preparatory adjunct to conventional phantom-head training, has not been fully established.

The present study aimed to contribute to this area by evaluating the effect of integrating HVRS before conventional phantom-head training in access cavity preparation. In addition, students' perceptions of the HVRS experience were explored to provide complementary insight into its educational value. We hypothesized that prior HVRS training would enhance student performance in endodontic access cavity preparation compared with conventional training alone.

## 2 Material and methods

### 2.1 Ethics approval

This study was approved by the Research Committee of the Universidad Europea (Approval No. 2025-128). All participants provided written informed consent prior to enrollment. The consent form included information on the study objectives, voluntary participation and data confidentiality and anonymity, in accordance with institutional and ethical standards.

### 2.2 Sample size

The sample size was calculated using the GRANMO online calculator (https://www.datarus.eu/ca/aplications/granmo/), applying the formula for comparing two independent means and based on previously published results (13). Assuming a two-sided alpha level of 0.05, a statistical power of 80%, an expected difference of 1.2 units, and a common standard deviation of 1.3, the minimum required sample size was 19 participants per group. To account for potential dropouts, the target was increased to 20 students per group.

### 2.3 Study design

This was a single-center, quasi-experimental, controlled study with stratified allocation, conducted during the 2024–2025 academic year at the Universidad Europea de Valencia. Outcome evaluators were blinded to group allocation.

### 2.4 Participants and eligibility

The target population consisted of fourth-year dental students enrolled in the *Restorative III/IV* course sequence. An email invitation outlining the study design and objectives was sent to all eligible students. Inclusion criteria were: successful completion of the competency-based preclinical phantom-head training with artificial teeth embedded in the official curriculum from year 1 to year 4 (including the *Restorative* sequence), and no previous exposure to the Simodont® HVRS.

In our program, preclinical endodontic access training on phantom heads is introduced within *Restorative III*, where students complete supervised access exercises on multiple tooth types (incisor, premolar, molar). The present study was conducted in *Restorative IV* to assess performance on a standardized premolar task. At this stage, students had not yet performed clinical (patient) access procedures.

Before any study procedures, all participants attended a standardized theoretical lecture covering endodontic access principles (outline design, removal of the pulp chamber roof, and prevention of pulpal floor perforations). The lecture was delivered within the curriculum by an endodontics faculty member with more than six years of teaching experience, ensuring consistency in content delivery. Attendance was mandatory and verified.

Exclusion criteria were: failure to complete the phantom-based preclinical training, or prior clinical or simulation experience in endodontic access cavity preparation outside the regular curriculum.

Participants were first ranked according to their prior preclinical performance (grade in the supervised endodontic access exercises within *Restorative III*). To ensure baseline balance between groups, a stratified allocation method was applied: students were alternately assigned to the experimental or control group in a sequential order (odd- vs. even-numbered ranks). Because simple randomization was not feasible, we applied stratified allocation by prior preclinical performance to enhance group comparability.

### 2.5 Interventions

In the experimental group, students first performed three simulated access cavity preparations using the Simodont® haptic simulator (Nissin Dental Products Europe B.V., Nieuw-Vennep, Netherlands; software version v4.22). The virtual exercise provided 3D visualization of the tooth and pulp chamber, tactile feedback simulating bur resistance, and auditory cues replicating handpiece sound. Students practiced outline design, roof removal, and internal form on a virtual premolar (#14) following the manufacturer's protocol. After completing this module, students proceeded to conventional training and prepared an access cavity on an artificial premolar mounted in a phantom head.

In the control group, students directly completed the conventional access cavity preparation training by performing the procedure on an artificial premolar mounted in a phantom head, without prior exposure to haptic simulation. After all study assessments were completed, control students were offered HVRS practice.

In both groups, artificial premolars (Real-T Endo, Acadental, Inc., USA) were mounted on modular typodont (ModuPRO Endo, Acadental Inc., KS, EE. UU.) and placed in Frasaco® mannequins. Students could adjust the position of the mannequin during the access opening according to their needs. Burs included Endo Z-FG and 801l FG ball-shaped diamond burs (Dentsply Maillefer).

Both the HVRS and phantom-head tasks were completed in a self-paced manner, without time restrictions, to reflect typical preclinical training conditions and allow students to work at their own rhythm.

### 2.6 Outcome assessment

**Figure 1:**
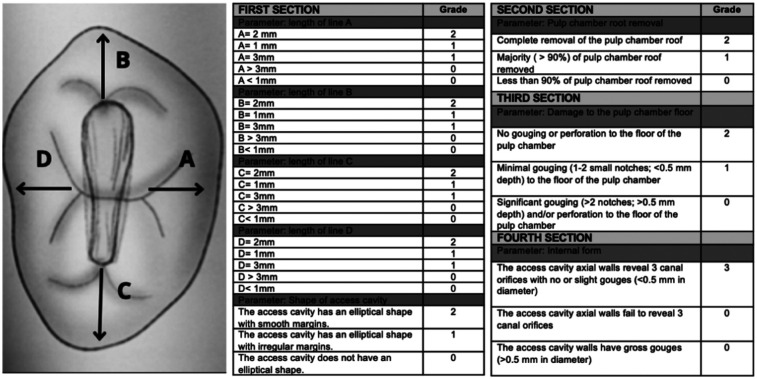
Rubric for evaluation of access cavity preparations, adapted from Slaczka et al. (14).

The final access cavity preparations were independently assessed by two calibrated evaluators, blinded to group allocation. A structured rubric adapted from Slaczka et al. (14) was used to evaluate four sections (Figure 1). The maximum cumulative score was 17 points. All specimens were anonymized and coded to prevent evaluator bias. Inter-rater reliability for each rubric item was calculated using the weighted Cohen's kappa coefficient to assess overall agreement across the rubric items.

### 2.7 Student satisfaction and perception

After completing the training with the Simodont®, only participants in the experimental group completed a structured satisfaction questionnaire. The survey included eight items rated on a 5-point Likert scale (1 = Strongly disagree to 5 = Strongly agree), covering perceived usefulness, realism, and overall satisfaction with the haptic VR training. Additionally, students indicated their preferred training modality (artificial teeth only, HVRS only, or a combination of both). All responses were anonymous and stored in a secure database for analysis. After study completion, some control students later used HVRS within routine teaching; no *post hoc* questionnaire was administered to them.

### 2.8 Statistical analysis

Normality of data distribution was evaluated using the Kolmogorov–Smirnov test. Comparisons between groups were performed using Student's *t*-test for normally distributed data or the Mann–Whitney *U* test for non-parametric data, as appropriate. Descriptive statistics were used for the questionnaire responses, and frequency distributions were reported for preferred training modality. A *p* value < 0.05 was considered statistically significant. Quantitative results are reported as mean ± standard error of the mean (SEM). Statistical analysis were performed using GraphPad Software Inc. (San Diego, CA, USA).

## 3 Results

### 3.1 Participants flow and group allocation

**Figure 2:**
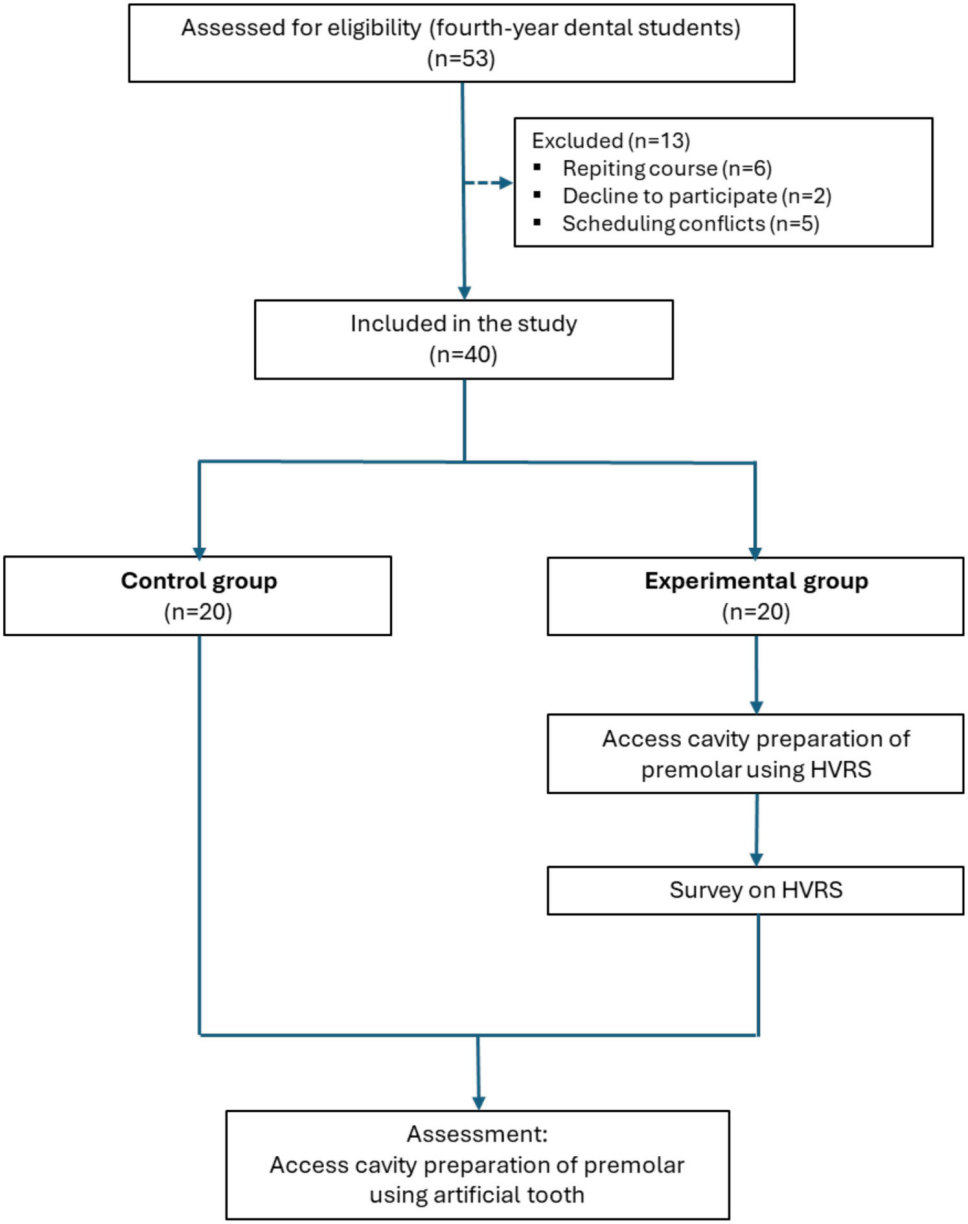
Flow chart showing the sequential phases of the study.

A total of 53 students expressed interest in participating. Thirteen were excluded due to predefined criteria (6 repeating the course, 5 unable to attend scheduled sessions, and 2 who declined participation), resulting in a final study sample of 40 students (13 male and 27 female, aged 22–23). Following ranked allocation based on prior preclinical performance, 20 students were assigned to the control group and 20 to the experimental group (Figure 2).

### 3.2 Performance outcomes

Access cavity preparations were independently evaluated by two calibrated evaluators. The overall inter-rater agreement, calculated using the weighted Cohen's kappa coefficient, was 0.89, indicating strong consistency between evaluators.

**Figure 3:**
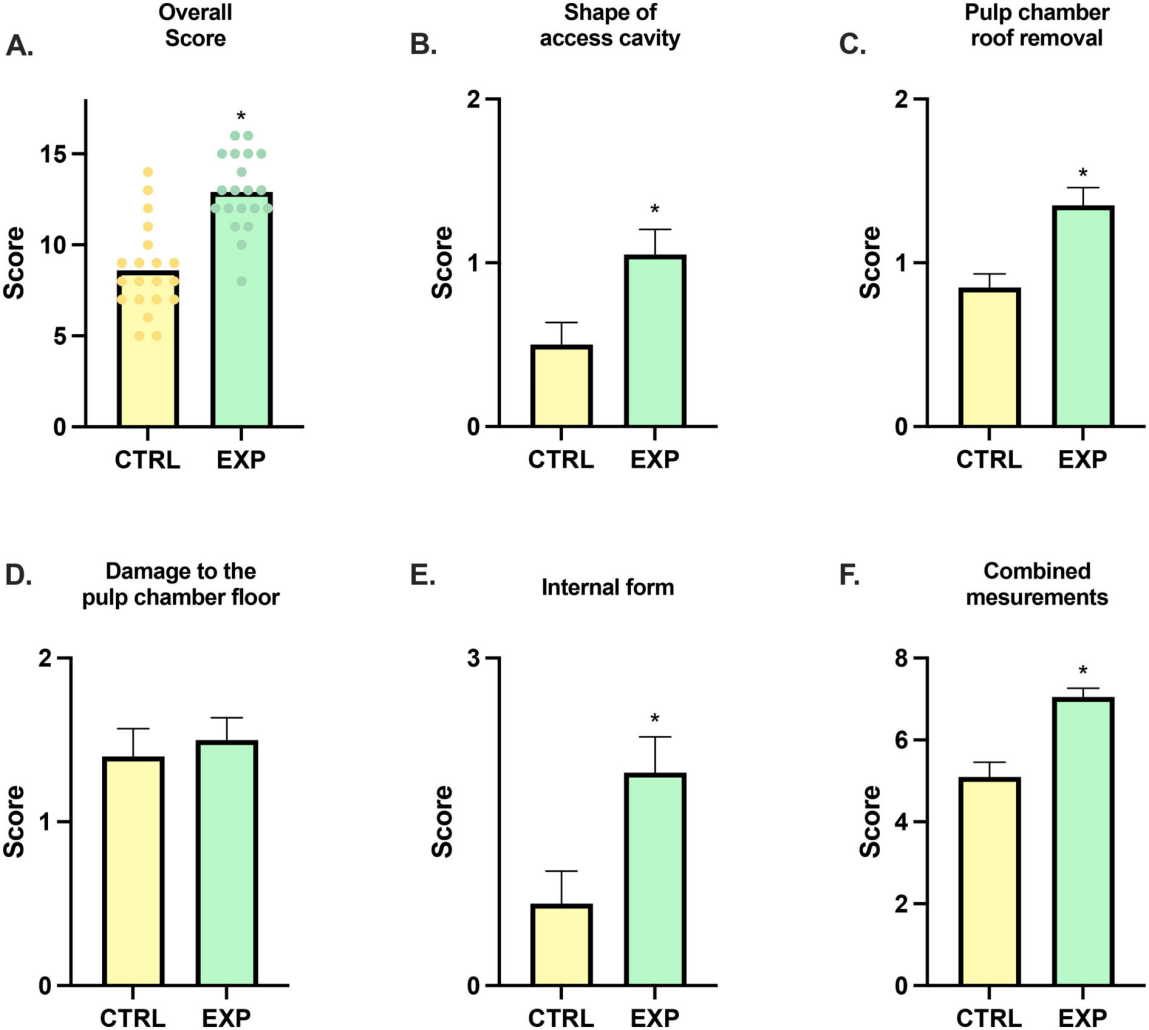
Comparative scores for endodontic access cavity preparations performed by the control group (CTRL) and experimental group (EXP). **(A)** Overall score. **(B)** Shape of access cavity. **(C)** Pulp chamber roof removal. **(D)** Damage to the pulp chamber floor. **(E)** Internal form. **(F)** Combined linear measurements **(A–D)** sum of scores from the first section of the rubric. All data are expressed as mean values ± standard error of the mean (SEM). The asterisk (*) indicates statistically significant differences compared with the control group, with a significance level set at *p* < 0.05.

All participants completed the assigned training and assessment. Performance scores obtained using the structured rubric were significantly higher in the experimental group compared to the control group. Improvements were observed in the overall score, shape of the access cavity, pulp chamber roof removal, internal form, and the combined linear measurements (sum of scores from section one: Lengths A–D). All between-group differences were statistically significant (*p* < 0.05). No significant difference was observed for the item “Damage to the pulpal floor” (*p* > 0.05) (Table 1, Figure 3).

**Table 1:** Comparison of performance scores for access cavity preparation between the control group (CTRL) and the experimental group (EXP), based on the structured rubric.

Rubric item	CTRL	EXP	*p*-value
Overall score	8.6 ± 0.54	12.9 ± 0.46	<0.01
Shape of access cavity	0.5 ± 0.14	1.05 ± 0.15	0.01
Pulp chamber roof removal	0.85 ± 0.08	1.35 ± 0.11	<0.01
Damage to the pulp chamber floor	1.4 ± 0.17	1.5 ± 0.14	0.65
Internal form	0.75 ± 0.30	1.95 ± 0.33	0.01
Combined linear measurements (A–D)	5.1 ± 0.35	7.05 ± 0.21	<0.01

Data are presented as mean ± standard error of the mean (SEM). Statistical comparisons were performed using unpaired Student's *t*-test. A *p*-value <0.05 was considered statistically significant.

### 3.3 Student satisfaction and perception

Students in the experimental group (*n* = 20) reported varied experiences with the Simodont® simulator. A majority (85%) agreed that it improved their ability to visualize the three-dimensional anatomy of the pulp chamber. Additionally, 70% enjoyed the simulator experience, 65% stated they would recommend it to peers, and 60% found the simulator easy to adapt to.

**Figure 4:**
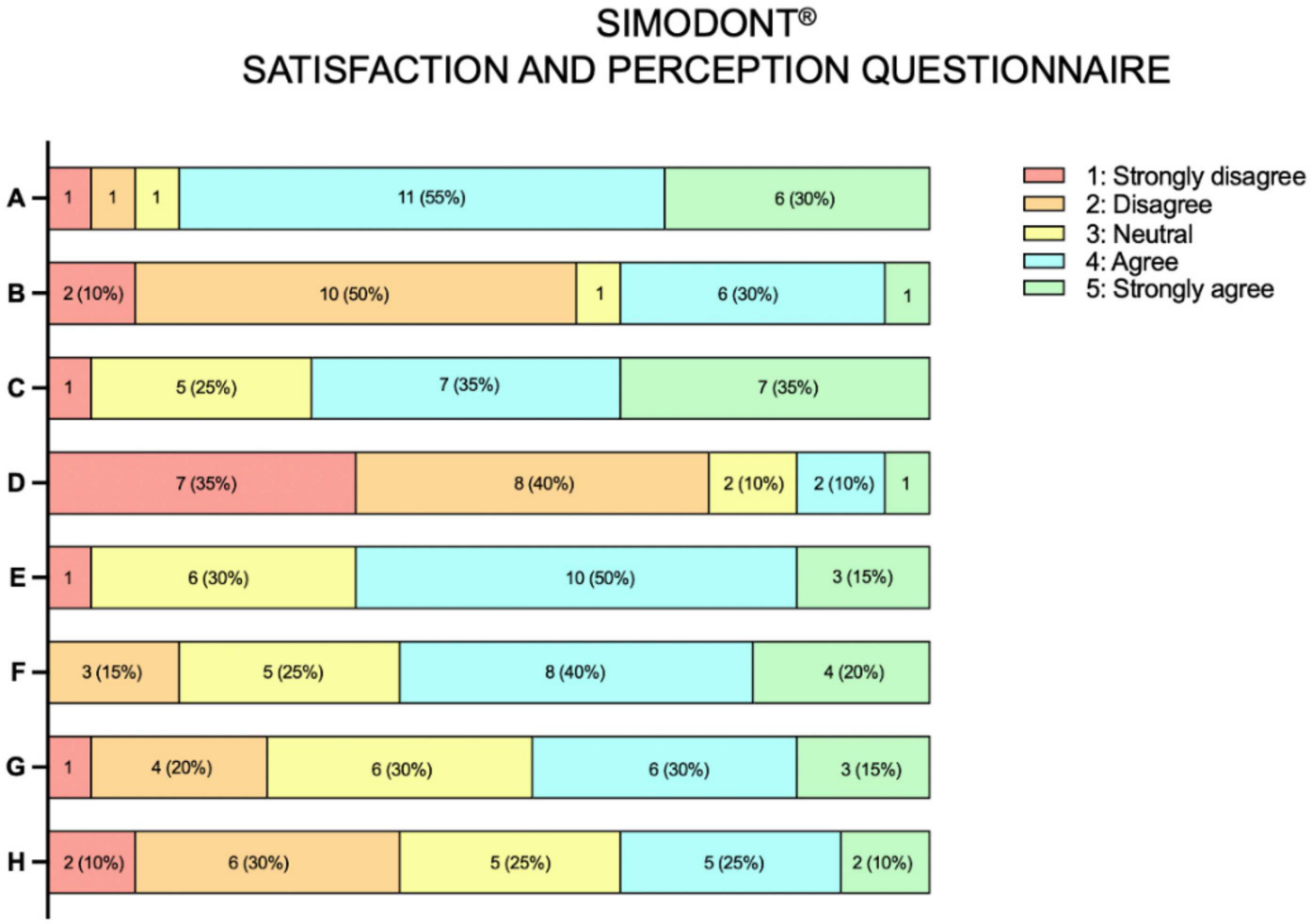
Responses to the satisfaction and perception questionnaire after simodont® simulator training (*n* = 20). **(A)** Simodont® helped me visualize the 3D anatomy of the pulp. **(B)** The haptic feedback of Simodont® was similar to that of a real handpiece. **(C)** I enjoyed using Simodont®. **(D)** I prefer Simodont® over artificial teeth. **(E)** I would recommend Simodont® to friends/colleagues. **(F)** It was easy to adapt to using Simodont®. **(G)** Using Simodont® has increased my confidence in performing dental procedures on patients. **(H)** The level of realism provided by Simodont® is sufficient to simulate real clinical scenarios.

Conversely, 60% disagreed that the haptic feedback resembled that of a real dental handpiece. Only 15% preferred using the simulator training over artificial teeth, 45% reported increased confidence in performing dental procedures after simulation, and 35% considered the simulator sufficiently realistic to replicate clinical scenarios (Figure 4).

**Figure 5:**
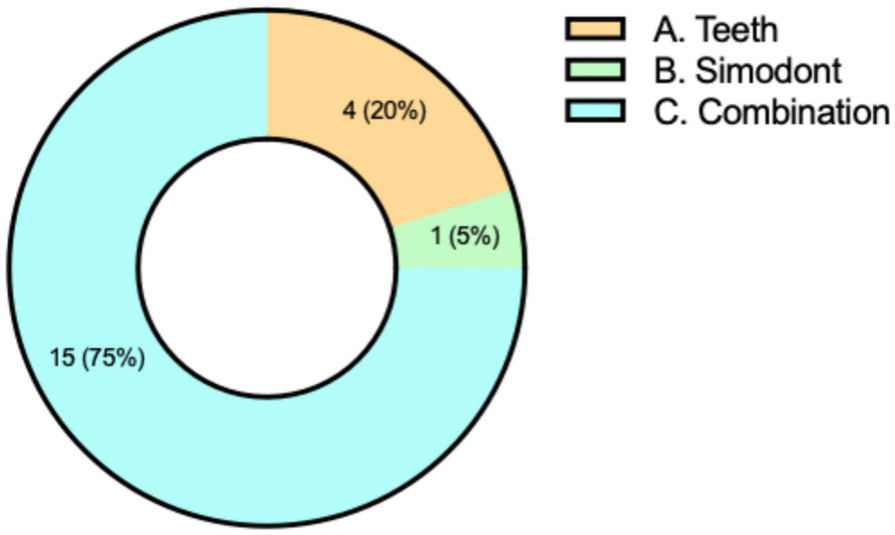
Participants' preferences for preclinical endodontic training modality. Responses to the multiple-choice item regarding preferred training method: **(A)** artificial teeth. **(B)** Simodont®. **(C)** Combination of both modalities. Values are shown as number of responses and percentage of the total sample (*n* = 20).

When asked about preferred training formats, 75% of participants in the experimental group selected a hybrid approach combining haptic simulation and artificial teeth. In comparison, 20% preferred training with artificial teeth alone, and only 5% favored the exclusive use of the simulator (Figure 5).

## 4 Discussion

This study examined the effect of integrating HVRS as a preparatory phase prior to conventional training in endodontic access cavity preparation. Students in the experimental group obtained significantly higher scores in overall performance, cavity shape, roof removal, and internal form, supporting our hypothesis that prior exposure to HVRS enhances technical accuracy when performing conventional preclinical training. These findings suggest that structured haptic simulation may reinforce spatial orientation and procedural control, allowing students to transfer skills more effectively to traditional models.

From an endodontic perspective, improvements in access cavity shape and internal form are particularly relevant, as these factors are directly linked to the ability to localize canal orifices, maintain straight-line access, and preserve tooth structure (3–5). Adequate roof removal further contributes to proper visualization of the pulp chamber and may prevent missed canals or iatrogenic complications. Consistent with contemporary minimally invasive principles, accurate and conservative access has become a central educational objective given its influence on instrumentation efficacy and fracture resistance across tooth types (15).

The use of premolars as the test teeth deserves consideration. While incisors might be considered more straightforward for a first attempt, premolars were intentionally selected because their morphology represents a greater challenge and therefore provides a more rigorous test of technical performance. This choice enhances the external validity of the findings, as it better reflects the complexity of clinical scenarios students will face.

Our results are consistent with previous findings showing that VR training may improve manual dexterity, coordination, and procedural accuracy. Suebnukarn et al. (11) and Hsu et al. (16) reported that simulation enhanced skill acquisition and reduced technical errors, while Yang and Chang (17) observed that training with Simodont® improved psychomotor control and student confidence. Similarly, the higher scores observed in our experimental group may be interpreted as a consequence of improved spatial orientation and motor control acquired during the preparatory HVRS sessions.

When comparing our results with existing literature, it is noteworthy that Usta et al. (13) also investigated preclinical endodontic training and confirmed the benefits of HVRS. Although their protocol differed, since students in the control group were later allowed to use the simulator to examine exposure time, both studies support the idea that HVRS contributes positively to student outcomes. Likewise, Slaczka et al. (14) did not find significant differences when HVRS and conventional methods were applied as separate alternatives, which underscores the importance of instructional sequencing. In addition, Suebnukarn et al. (18) observed comparable reductions in technical errors across both HVRS and traditional training modalities, suggesting that the benefits of simulation may vary depending on instructional design and evaluation methods. The present results reinforce that HVRS may be most beneficial when integrated sequentially as a preparatory step rather than applied in isolation. From an implementation standpoint, embedding haptics-enhanced VR into routine teaching entails both benefits (skill acquisition, engagement) and challenges (costs, faculty development, workflow integration, fidelity constraints) that should be anticipated during curricular planning (19).

Beyond performance, student perceptions provided complementary insights. Most participants valued HVRS as a useful tool for visualizing the three-dimensional anatomy of the pulp chamber and appreciated the possibility of practicing in a risk-free environment. These perceptions are consistent with previous reports where students highlighted VR-based training as helpful for spatial awareness and conceptual understanding (14, 20). However, limitations in tactile fidelity were also evident, as a majority of students disagreed that the haptic sensation resembled a real handpiece. Similar concerns have been reported in the literature, including limited ergonomics and depth perception (21, 22). Importantly, the preference of most students for a hybrid training model combining HVRS with artificial teeth aligns with recent multicenter findings emphasizing the complementary, rather than substitutive, role of simulation (6).

The absence of significant differences in pulpal floor damage may be explained by depth control being less influenced by spatial visualization and more by tactile fidelity, which remains limited in current HVRS systems. Similarly, dissatisfaction regarding haptic realism highlights a technological constraint that future developments may overcome.

HVRS sits within a wider spectrum of technology-enhanced learning that includes immersive and mixed-reality systems. Recent studies have demonstrated that immersive reality can enhance surgical training in complex procedures such as orthognathic surgery (23), while mixed reality has shown advanced outcomes in guiding operative steps and improving three-dimensional understanding (24). Furthermore, VR has been successfully applied to reduce anxiety and improve hemodynamic control in patients undergoing oral surgery under local anesthesia (25). Although these modalities differ in their degree of immersion and interaction, together they highlight a growing movement toward technology-enhanced learning environments. HVRS fits within this continuum by providing tactile feedback and procedural realism, complementing the visual immersion of other systems. Future studies may benefit from exploring how haptic simulation can be combined with immersive or mixed reality to create integrated training models that maximize both cognitive and psychomotor learning.

Despite these positive outcomes, several limitations should be acknowledged. This single-centre study with a relatively small sample may limit generalizability. The design was quasi-experimental with stratified allocation by prior performance rather than randomized, so residual confounding cannot be excluded despite standardized prior instruction and assessor blinding. Only premolars were tested, and performance might differ with other tooth types. Finally, although evaluators were calibrated, assessment relied on preclinical models and did not extend to clinical outcomes. Although the inclusion of HVRS may have increased student engagement, this reflects real-world multimodal teaching strategies; future studies may consider designs that isolate engagement effects from simulator impact. These limitations suggest caution in extrapolating results and highlight the need for multicenter studies with larger cohorts and clinical endpoints.

## 5 Conclusions

This study suggests that the integration of HVRS as a preparatory phase prior to conventional training enhanced student performance in endodontic access cavity preparation and may represent a valuable complement to traditional teaching methods.

## Data Availability

The raw data supporting the conclusions of this article will be made available by the authors, without undue reservation.

## References

[1] Ba-Hattab R Taha NA Shaweesh MM Palma PJ Abdulrab S. Global trends in preclinical and clinical undergraduate endodontic education: a worldwide survey. Sci Rep. (2025) 15(1):10078. 10.1038/s41598-025-94836-y 40128271 PMC11933302

[2] Luz LB Grock CH Oliveira VF Bizarro L Ardenghi TM Ferreira MBC Self-reported confidence and anxiety over endodontic procedures in undergraduate students-quantitative and qualitative study. Eur J Dent Educ. (2019) 23(4):482–90. 10.1111/eje.12456 31373094

[3] Clark D Khademi J. Modern molar endodontic access and directed dentin conservation. Dent Clin North Am. (2010) 54(2):249–73. 10.1016/j.cden.2010.01.001 20433977

[4] Plotino G Grande NM Isufi A Ioppolo P Pedullà E Bedini R Fracture strength of endodontically treated teeth with different access cavity designs. J Endod. (2017) 43(6):995–1000. 10.1016/j.joen.2017.01.022 28416305

[5] Krastl G Zehnder MS Connert T Weiger R Kühl S. Guided endodontics: a novel treatment approach for teeth with pulp canal calcification and apical pathology. Dent Traumatol. (2016) 32(3):240–6. 10.1111/edt.12235 26449290

[6] Serrano CM Bakker DR Zamani M de Boer IR Koopman P Wesselink PR Virtual reality and haptics in dental education: implementation progress and lessons learned after a decade. Eur J Dent Educ. (2023) 27(4):833–40. 10.1111/eje.12873 36367342

[7] Patil S Bhandi S Awan KH Licari FW Di Blasio M Ronsivalle V Effectiveness of haptic feedback devices in preclinical training of dental students-a systematic review. BMC Oral Health. (2023) 23(1):739. 10.1186/s12903-023-03410-3 37817151 PMC10566064

[8] Bandiaky ON Lopez S Hamon L Clouet R Soueidan A Le Guehennec L. Impact of haptic simulators in preclinical dental education: a systematic review. J Dent Educ. (2024) 88(3):366–79. 10.1002/jdd.13426 38044266

[9] Al-Saud LM. The utility of haptic simulation in early restorative dental training: a scoping review. J Dent Educ. (2021) 85(5):704–21. 10.1002/jdd.12518 33368289

[10] Koolivand H Shooreshi MM Safari-Faramani R Borji M Mansoory MS Moradpoor H Comparison of the effectiveness of virtual reality-based education and conventional teaching methods in dental education: a systematic review. BMC Med Educ. (2024) 24(1):8. 10.1186/s12909-023-04954-2 38172742 PMC10765860

[11] Suebnukarn S Haddawy P Rhienmora P Gajananan K. Haptic virtual reality for skill acquisition in endodontics. J Endod. (2010) 36(1):53–5. 10.1016/j.joen.2009.09.020 20003935

[12] Suebnukarn S Haddawy P Rhienmora P Jittimanee P Viratket P. Augmented kinematic feedback from haptic virtual reality for dental skill acquisition. J Dent Educ. (2010) 74(12):1357–66. 10.1002/j.0022-0337.2010.74.12.tb05011.x 21123503

[13] Usta SN Silva EJNL Keskin C Tekkanat H Liukkonen M Felszeghy S. A comparison of traditional and virtual reality haptic simulator approaches in preclinical endodontic training: impacts on skill acquisition, confidence and stress. Int Endod J. (2025). 10.1111/iej.14236 PMC13158535 40207994

[14] Slaczka DM Shah R Liu C Zou F Karunanayake GA. Endodontic access cavity training using artificial teeth and Simodont® dental trainer: a comparison of student performance and acceptance. Int Endod J. (2024). 10.1111/iej.14171 39555944

[15] Krishan R Paqué F Ossareh A Kishen A Dao T Friedman S. Impacts of conservative endodontic cavity on root canal instrumentation efficacy and resistance to fracture assessed in incisors, premolars, and molars. J Endod. (2014) 40(8):1160–6. 10.1016/j.joen.2013.12.012 25069925

[16] Hsu MH Liu CM Chen CJ Yang HW Chang YC. Virtual 3D tooth creation for personized haptic simulation training in access cavity preparation. J Dent Sci. (2022) 17(4):1850–3. 10.1016/j.jds.2022.06.014 36299325 PMC9588804

[17] Yang PY Chang YC. The haptic 3D virtual reality dental training simulator as a good educational tool in preclinical simulation learning. J Dent Sci. (2022) 17(1):618–9. 10.1016/j.jds.2021.10.016 35028103 PMC8740112

[18] Suebnukarn S Hataidechadusadee R Suwannasri N Suprasert N Rhienmora P Haddawy P. Access cavity preparation training using haptic virtual reality and microcomputed tomography tooth models. Int Endod J. (2011) 44(11):983–9. 10.1111/j.1365-2591.2011.01899.x 21623838

[19] Felszeghy S Mutluay M Liukkonen M Flacco N Bakr MM Rampf S Benefits and challenges of the integration of haptics-enhanced virtual reality training within dental curricula. J Dent Educ. (2024) 89(7):1070–83. 10.1002/jdd.13800 39690427 PMC12268116

[20] Philip N Ali K Duggal M Daas H Nazzal H. Effectiveness and student perceptions of haptic virtual reality simulation training as an instructional tool in pre-clinical paediatric dentistry: a pilot pedagogical study. Int J Environ Res Public Health. (2023) 20(5):4226. 10.3390/ijerph20054226 36901241 PMC10001601

[21] Huang Y Huang S Liu Y Lin Z Hong Y Li X. Application of virtual reality and haptics system Simodont in Chinese dental education: a scoping review. Eur J Dent Educ. (2023) 29(3):585–93. 10.1111/eje.12984 38148502

[22] Hattori A Tonami K-i Tsuruta J Hideshima M Kimura Y Nitta H Effect of the haptic 3D virtual reality dental training simulator on assessment of tooth preparation. J Dent Sci. (2022) 17(1):514–20. 10.1016/j.jds.2021.06.022 35028078 PMC8740096

[23] Stevanie C Ariestiana YY Anshar M Sukotjo C Boffano P Forouzanfar T Immersive reality surgical training for Le Fort I orthognathic surgery: initial results of a randomized feasibility study. J Craniomaxillofac Surg. (2025) 53(7):1009–17. 10.1016/j.jcms.2025.03.014 40189948

[24] Stevanie C Ariestiana YY Hendra FN Anshar M Boffano P Forouzanfar T Advanced outcomes of mixed reality usage in orthognathic surgery: a systematic review. Maxillofac Plast Reconstr Surg. (2024) 46(1):29. 10.1186/s40902-024-00440-x 39073682 PMC11286605

[25] Valls-Ontañón A Vandepputte SS de la Fuente C Giralt-Hernando M Molins-Ballabriga G Cigarrán-Mensa M Effectiveness of virtual reality in relieving anxiety and controlling hemodynamics during oral surgery under local anesthesia: a prospective randomized comparative study. J Craniomaxillofac Surg. (2024) 52(3):273–8. 10.1016/j.jcms.2024.01.021 38326127

